# Competency-based training in anaesthesiology: train the trainers first. A descriptive cross-sectional survey by the committee of the European Society of Anaesthesiology and Intensive Care (ESAIC) Teach the Teachers Masterclass

**DOI:** 10.3389/fmed.2024.1512308

**Published:** 2025-02-03

**Authors:** Vojislava Neskovic, Carmen Oliveira, Aeyal Raz, Lesley Bromley, Gordana Jovanovic

**Affiliations:** ^1^Military Medical Academy, Faculty of Medicine of MMA, Belgrade, Serbia; ^2^Center for Health Technology and Services Research–CINTESIS, Faculty of Medicine, Porto University, Porto, Portugal; ^3^Department of Anaesthesiology, Centro Hospitalar de Vila Nova de Gaia/Espinho, Vila Nova de Gaia, Portugal; ^4^Department of Anesthesia, Rambam Health Care Campus and The Ruth and Bruce Rappaport Faculty of Medicine, Technion e Israel Institute of Technology, Haifa, Israel; ^5^Consultant Emeritus University College Hospital NHS Foundation Trust, London, United Kingdom; ^6^Faculty of Medicine of the University of Novi Sad, Novi Sad, Serbia

**Keywords:** survey, training of trainers, anaesthesiology, education, medical

## Abstract

**Introduction:**

The level of competence in teaching among trainers expected to deliver training according to the European Training Requirement in anaesthesiology is unknown. The aim of this descriptive cross-sectional survey, performed from 1 September 2021 until 31 October 2021, and promoted by the European Society of Anaesthesiology and Intensive Care (ESAIC) was to establish the current level of knowledge and faculty development among European countries regarding competency-based education and training (CBMET) in anesthesia and intensive care.

**Results:**

A total of 711 responses of anaesthesiologists working in 46 European countries were analyzed. The great majority (530/74.64%) had more than 10 years of experience in anesthesia, were experienced specialists, or held more senior positions (645/90.97%), worked in academic hospitals (451/63.5%), and claimed to be involved in teaching residents (561/79.01%). Most respondents declared either not sure or no knowledge (115/546; 21.06% and 232/546; 42.49%) about European training requirements in anaesthesiology. One-third claim to know about CBMET. Formal training in teaching has 21% of respondents. Lack of time (369/506; 72.92%) and overload with work (351/506; 69.36%) are reported as the most important obstacles in teaching residents. A disparity in the answers is present between, but within the countries too.

**Conclusions:**

The results of the presented survey reveal that even among experienced anesthesia professionals dedicated to medical education there is still a lack of knowledge on CBMET as well as systemic support for faculty development in European countries. The differences within and between European countries regarding the perception of CBMET. Dedication to faculty development is necessary to improve European anesthesia and intensive care education.

## Introduction

The first guidelines for competence-based postgraduate training in anesthesia, pain, and intensive care were published years ago (1). They recognize the set of skills, knowledge, and attitudes that modern anaesthesiologists are expected to possess and master to perform perioperative management of surgical patients successfully and safely. Updates have been available, and the last European Training Requirement (ETR) in Anesthesiology Update 2022, from the Standing Committee on Education and Professional Development (EPD) of the section and European Union of Medical Specialists (UEMS), Board of Anesthesiology (EBA) was published recently (2).

Although there are differences between countries in technology, organization, infrastructure, workforce, or standard of care, it is well assumed that the introduction of competency-based education and training (CBMET) may set a common ground for all of them. Undergraduate medical education has already included learning outcomes harmonization based on the Bologna Declaration and process (3). With that experience in mind, once training is set as outcome-based, traditional educational programs and syllabuses must change too (3, 4). New teaching programs and styles, teaching facilities and learning opportunities, changed curricula, but even more importantly, advanced assessment strategies of acquired competencies are necessary (4, 5).

It is well-recognized that the role of the teacher or a trainer must be changed toward more complex performance: facilitator, role model, assessor, planner, information provider, or resource developer (6). Trainers need more advanced competencies to implement and achieve objectives from the competence-based training curricula: knowledge and skills, support, but the most demanding requirement is the time dedicated to delivering educational content (7). One of the prerequisites in implementing CBMET is that trainers understand the theory behind the new education model and train themselves to teach within the new framework (8).

Literature and previous experiences in implementing new training suggest that trainers are insufficiently prepared to employ active learning strategies, and instead of effectively delivering more advanced programs tend to set back to previous and known types of teaching (7, 9). Faculty often lack formal training and understanding of CBMET and have no skill in assessment resulting in inconsistencies in judgment and expectations of learner's performance (10, 11).

ETR in anaesthesiology implies that CBMET is accepted as the new standard (2).

A competency-based curriculum clearly defines the tasks trainees are expected to perform and the methods by which their competence will be assessed, potentially facilitating the comparison of programs (12). The current framework includes Entrustable Professional Activities (EPAs). An EPA is a key task in the profession that trainees can be trusted to perform unsupervised once they have shown they can do it competently. By the end of the training program, it should be clear which areas of anesthesia a graduate is trusted to handle independently (12).

Today's trainers have been educated and trained in a time- or count-based environment, but they are expected to create training environments and teaching programs for CBMET. The European Training Requirements (ETR) document is supported by a handbook outlining teaching, assessment, and feedback methods to assist trainers in meeting these expectations (2). However, the level of teaching knowledge among trainers in European countries involved in education remains unclear, and it is uncertain how these programs will be effectively delivered.

The objective of this survey was to establish the current level of knowledge and faculty development among European countries regarding CBMET in anesthesia and intensive care.

## Methods

Between 2013 and 2022, the European Society of Anaesthesiology and Intensive Care (ESAIC) organized 9 consecutive Train the Trainer (TTT) courses, each consisting of 2 weeks of interactive teaching with a 6-month practice period in between. These courses aimed to develop personal teaching skills and introduce foundational knowledge of medical education. The target participants were final-year residents or recently graduated young specialists in anesthesia and intensive care from European countries who were active members of ESAIC. While the course content, later known as the Masterclass, evolved to reflect the current level of knowledge and participant expectations, feedback from both faculty and graduates indicated a significant gap in training and the status of anesthesia trainers across different educational environments and countries. To address this, faculty members who are representatives in the ESAIC TTT Masterclass Committee conducted a descriptive cross-sectional survey to gather information on the existing knowledge and standards for training anesthesia trainers in CBMET among ESAIC's active members.

The ESAIC TTT faculty and committee members developed a questionnaire of 30 questions to collect the initial data. The focus was on the existing background and common practice in teaching, formal training in medical education, actual knowledge of competence-based curricula, and perceived barriers to performing teaching. The questions were allocated into three groups: general and professional data, involvement and experience in teaching residents, and an overview of the anesthesia curriculum within the country of work. Questions on teaching experience were open to respondents who stated they were involved in teaching residents. Content validation and pretesting were performed twice by the TTT Faculty and invited graduated students from previous TTT Masterclass courses. The final version in English (**Supplemental Digital Content** – link to the survey) was created and obtained ESAIC Board approval.

An emailed link was sent to ESAIC's list of members on 1 September 2021. Email list which was used to send the survey link was the official mailing list of all anesthesia professionals practicing in European countries, who are listed as active members of the ESAIC. Reminders have been emailed within 2 months (after 1 month and after every 15 days) from the official ESAIC communication email address. The survey was promoted on ESAIC social media (Facebook) to raise attention to the society members to respond to the official email invitation where the link to survey was included. Responders could only answer once. There were no exclusion criteria.

The reporting of this study followed the STROBE guidelines.

### Ethics

Since objectives and information about the further use of data were clearly stated, it was assumed that filling out the questionnaire was consent for participation. However, the survey was presented to the Ethical Board of Centro Hospitalar de Vila Nova de Gaia/Espinho which has waived the need for informed consent (document 06/2023-1). The survey was anonymous, and the information confidential. All data are stored on ESAIC's server.

### Bias

Non-representative responses may be present from several countries due to non-response bias. Selection bias may be present since it was perceived that the topic is irrelevant to anesthetists not dedicated to medical education and teaching.

### Statistical analysis

Descriptive statistical analysis was performed, and variables were presented as numbers (n) and proportions (%). Respondents were allowed no answer or to give more than one response to the questions, and all reported proportions were relative to the number of responses to each question. Some data was presented as tables.

## Results

### Respondents experience in teaching

A total of 711 respondents were included in the analysis, representing an estimated response rate of 11.5% (out of 6,185 ESAIC members as of September 2021). Most respondents were based in 40 European countries (listed by the United Nations). Additionally, several respondents (12) were from Israel, an ESAIC Council member country, and one respondent each came from Uzbekistan, Kazakhstan, and Azerbaijan, all of whom were from the listed emails of active ESAIC members (individual membership).

Balanced number of female and male respondents were included (54% vs. 46%) and most of them were over 40 years of age (474/66.7%), had more than 10 years of experience in anesthesia (534/75.2%) and holds a specialist or more senior position at work (645/90.97%). Also, most respondents work in academic (university or teaching) hospitals (451/63.5%). Participants' demographics are presented in Table 1.

**Table 1:** Participants demographics.

**Data**		**Total *n* (%)**
Age	25–30	28 (4%)
	31–35	93 (13%)
	36–40	116 (16.3%)
	41–50	223 (31.4%)
	Over 50	251 (35.3%)
Gender	Female	384 (54%)
	Male	326 (46%)
	Non-binary	1
Years of practice	5–10	176 (24.8%)
	11–15	141 (19.9%)
	16–20	108 (15.2%)
	Over 20	285 (40.1%)
Current position	Trainee	34 (4.8%)
	Senior resident	34 (4.8%)
	Specialist	248 (35%)
	Lecturer	14 (2%)
	Senior specialist	203 (28.6%)
	Chief of department	94 (13.3%)
	Teacher university	64 (9%)
	Other	18 (2.5%)
Type of hospital	Academic	451 (63.5%)
	General	180 (25.4%)
	Specialized clinic	31 (4.4%)
	Private hospital	48 (6.7%)

A total of 79.01% of respondents (561/710) claimed to be involved in teaching residents. Out of those that teach, more than half (65.10%) teach residents every or almost every day and are involved in mentoring (65.21%).

The number of years in teaching, type of engagements, teaching skills used and involvement in different types of assessment (summative or formative) are presented in Table 2.

**Table 2:** Participants teaching data.

		**Total *n* (%)**
1. For how many years have you been teaching? (*n* = 505)	Less than 5	117 (23.2%)
	6–10	116 (23%)
	11–15	94 (18.6%)
	Over 16	178 (35.2%)
2. What are your engagements when teaching residents? (Choose all appropriate answers)	I give lectures	320 (63.4%)
	Perform work-based interactive teaching (operating room)	420 (83.1%)
	Running clinical case discussions	279 (55.2%)
	Involved in simulation sessions	205 (40.6%)
	Teach informally, during every day work	403 (79.8%)
	Other	29 (5.7%)
3. Which of the teaching skills do you use in your own teaching? (Choose all appropriate answers)	Planning and preparation for teaching session	318 (62.9%)
	Using visual aids	290 (57.4%)
	Interactive teaching	341 (67.5%)
	Problem based learning	330 (62.9%)
	Teaching the skills	388 (76.8%)
	Giving feedback	396 (78.4%)
	Organizing and leading the discussions	249 (49.3%)
	Organizing specific sessions (journal clubs, interactive sessions, pro and con debate…)	190 (37.6%)
	Other	8 (1.6%)
4. Are you involved in any form of assessment of the residents? (Choose all appropriate answers)	I am an examiner for the final exam	128 (25.3%)
	I am European Diploma in anaesthesia and Intensive Care (EDAIC) part II examiner	67 (13.2%)
	I occasionally carry out assessment during the residency program (Direct Observation of Procedural Skills–DOPS)	195 (38.6%)
	I give feedback during simulation sessions	202 (40%)
	I give feedback during everyday work	389 (77.0%)
	I am involved in other forms of formative assessment (for example multisource feedback)	168 (33.2%)
	I am not involved in any kind of assessment	53 (10.5%)
	Other	19 (3.8%)

Percentages refer to the total of participants that replied to these questions (*n* = 505). Questions 2, 3 and 4 allowed for more than one option.

The reasons for not being involved in teaching are employment in non-teaching or private hospitals (62/108; 57.4%). A small number of respondents (29/710; 4.08%) consider that everyday work with residents does not classify as teaching.

### Perceived barriers to performing teaching

Lack of time (369/506; 72.92%) and overload with work (351/506; 69.36%) are the most important reported obstacles in teaching, followed by lack of organization (200/506; 39.52%), lack of motivation (163/506; 32.21%) and lack of training (137/506; 27.08%). More than half (63%) of the respondents claim that their teaching is not valued in their departments at all or is considered a part of everyday work. Only 9.1% (53/581) claim that they are paid more as teachers. Regarding the value of teaching, responses differ between countries, but different and often conflicting answers come even from the same country. In United Kingdom of Great Britain and Northern Ireland out of 32 respondents 4 claim that they are paid more, 2 are more visible in the department and 6 are advanced in academic position due to teaching. However, 16 respondents claim that teaching is considered as a part of everyday work and one respondent stated that it is not valued at all. Similar proportion of responses comes from Germany (2 are paid more, 4 are promoted in academia,12 is more visible, 37 claim that it is a part of everyday work and 4 that it is not valued at all). On the other hand, in Albania teaching leads to better payment and promotion in academia (all 4 respondents).

### Formal training in medical education

**Figure 1:**
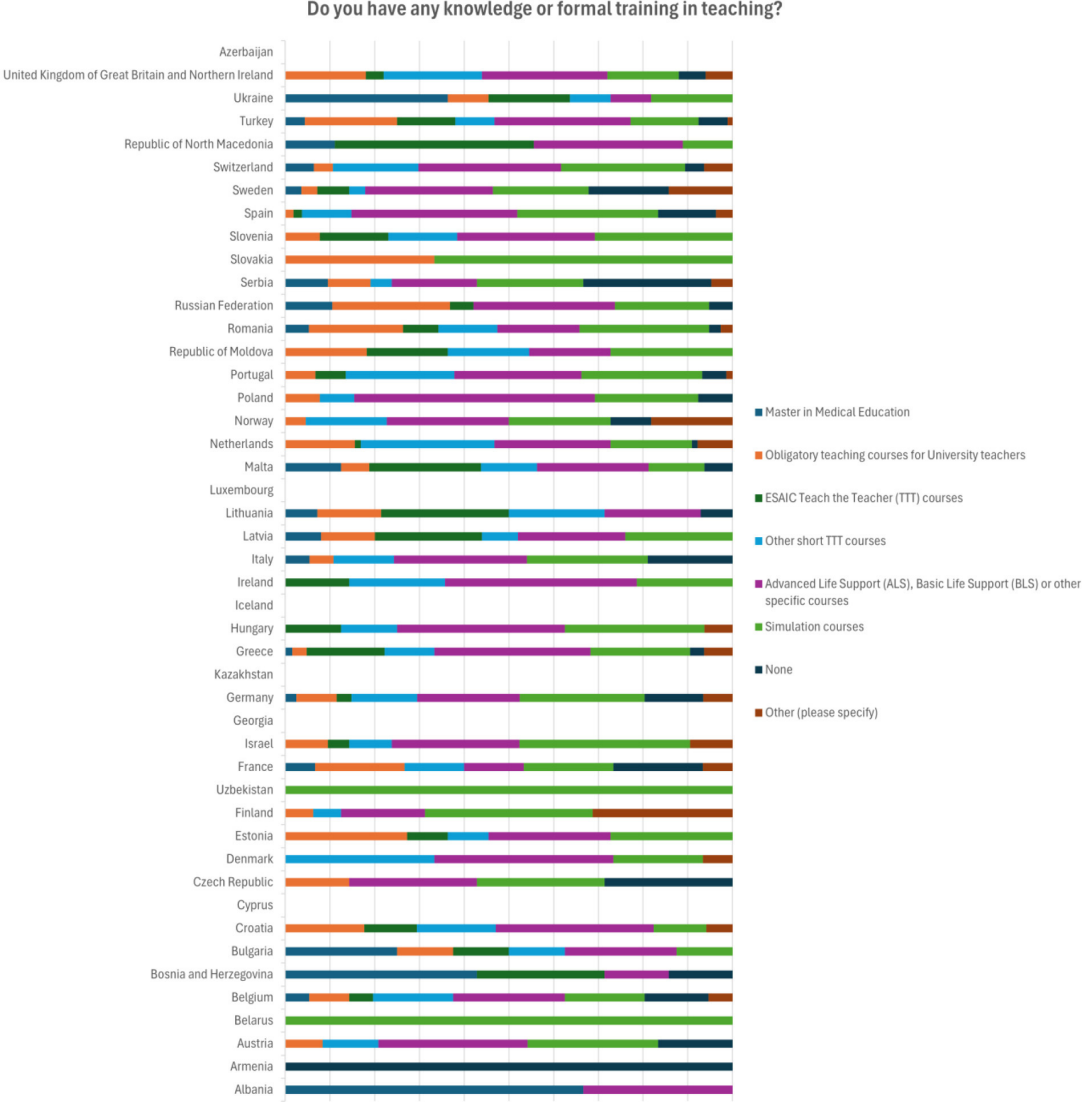
Distribution of answers by the country regarding the knowledge or formal training on teaching.

Formal training in teaching has only 21.7% of respondents (154/710). Short courses in teaching or ESAIC TTT courses were attended by 34% of respondents (242/710). Almost half (306/710 or 43.1%) recognize attending Advanced Life Support (ALS) and Basic Life Support (BLS) courses as training in teaching (Figure 1).

Mostly respondents from Germany, Netherlands, Turkey, and UK report having obligatory teaching courses for university teachers. At the same time, from Germany most respondents (23/68) report attending ALS and BLS courses as training in teaching and substantial number (16/68) report having no training in teaching at all (Table 3).

**Table 3:** Distribution of answers by the country regarding the knowledge or training on teaching.

**Do you have any knowledge or formal training in teaching?**	**Number of respondents**	**Master in medical education**	**Obligatory teaching courses for university teachers**	**ESAIC Teach the Teacher (TTT) courses**	**Other short TTT courses**	**Advanced life support (ALS), basic life support (BLS) or other specific courses**	**Simulation courses**	**None**	**Other (please specify)**
Albania	6	2	0	0	0	1	0	0	0
Armenia	1	0	0	0	0	0	0	1	0
Austria	15	0	2	0	3	8	7	4	0
Belarus	1	0	0	0	0	0	1	0	0
Belgium	30	3	5	3	10	14	10	8	3
Bosnia and Herzegovina	4	3	0	2	0	1	0	1	0
Bulgaria	3	2	1	1	1	2	1	0	0
Croatia	15	0	3	2	3	6	2	0	1
Cyprus	4	0	0	0	0	0	0	0	0
Czech Republic	5	0	1	0	0	2	2	2	0
Denmark	7	0	0	0	5	6	3	0	1
Estonia	3	0	3	1	1	3	3	0	0
Finland	8	0	1	0	1	3	6	0	5
Uzbekistan	1	0	0	0	0	0	1	0	0
France	14	1	3	0	2	2	3	3	1
Israel	12	0	2	1	2	6	8	0	2
Georgia	3	0	0	0	0	0	0	0	0
Germany	68	3	11	4	18	28	34	16	8
Kazakhstan	1	0	0	0	0	0	0	0	0
Greece	40	1	2	11	7	22	14	2	4
Hungary	6	0	0	2	2	6	5	0	1
Iceland	1	0	0	0	0	0	0	0	0
Ireland	8	0	0	2	3	6	3	0	0
Italy	30	2	2	0	5	11	10	7	0
Latvia	10	2	3	6	2	6	6	0	0
Lithuania	9	1	2	4	3	3	0	1	0
Luxembourg	1	0	0	0	0	0	0	0	0
Malta	7	2	1	4	2	4	2	1	0
Netherlands	35	0	12	1	23	20	14	1	6
Norway	11	0	1	0	4	6	5	2	4
Poland	15	0	1	0	1	7	3	1	0
Portugal	57	0	5	5	18	21	20	4	1
Republic of Moldova	4	0	2	2	2	2	3	0	0
Romania	25	2	8	3	5	7	11	1	1
Russian Federation	11	2	5	1	0	6	4	1	0
Serbia	31	2	2	0	1	4	5	6	1
Slovakia	3	0	1	0	0	0	2	0	0
Slovenia	7	0	1	2	2	4	4	0	0
Spain	49	0	1	1	6	20	17	7	2
Sweden	17	1	1	2	1	8	6	5	4
Switzerland	29	3	2	0	9	15	13	2	3
The former Yugoslav Republic of Macedonia	5	1	0	4	0	3	1	0	0
Turkey	58	4	19	12	8	28	14	6	1
Ukraine	7	4	1	2	1	1	2	0	0
United Kingdom of Great Britain and Northern Ireland	32	0	9	2	11	14	8	3	3
Azerbaijan	1	0	0	0	0	0	0	0	0
Sum	710	41	113	80	162	306	253	85	52

**Figure 2:**
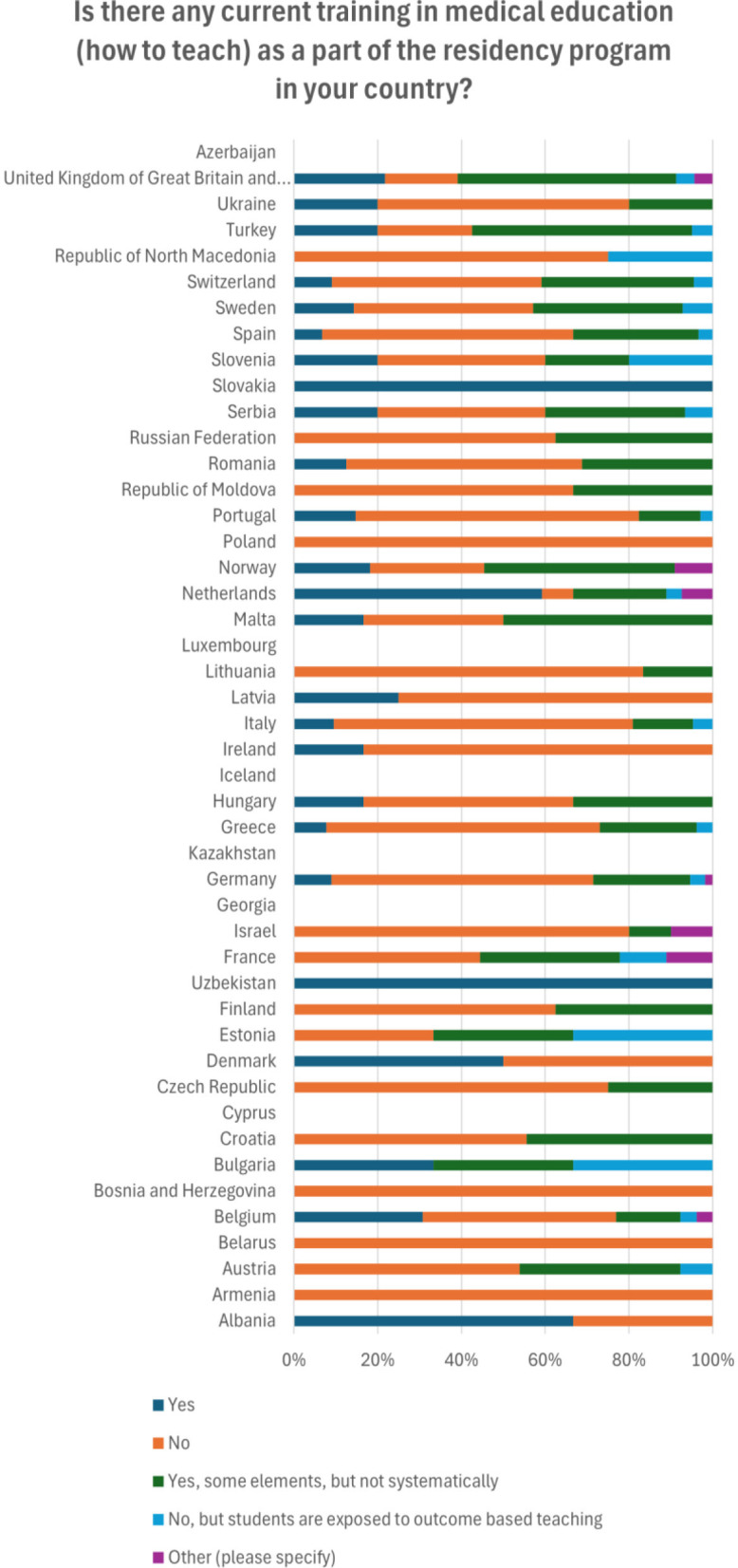
Distribution of answers by the countries regarding the training on teaching or medical education in the current residency curriculum.

Regarding training in medical education as a part of residency program, nearly half (258/504) deny that there is any, a minority (80/504) confirms there is training, while less than a third (138/504) reported there are some but not systematically applied elements of training (Figure 2). Netherland is the country with the highest number of respondents that claim that medical education is present during the residency program (16/35) (Table 4).

**Table 4:** Distribution of answers by the countries regarding the training on teaching or medical education in the current residency curriculum.

**Is there any current training in medical education (how to teach) as a part of the residency program in your country?**	**Number of respondents**	**Yes**	**No**	**Yes, some elements, but not systematically**	**No, but students are exposed to outcome based teaching**	**Other (please specify)**
Albania	6	2	1	0	0	0
Armenia	1	0	1	0	0	0
Austria	15	0	7	5	1	0
Belarus	1	0	1	0	0	0
Belgium	30	8	12	4	1	1
Bosnia and Herzegovina	4	0	4	0	0	0
Bulgaria	3	1	0	1	1	0
Croatia	15	0	5	4	0	0
Cyprus	4	0	0	0	0	0
Czech Republic	5	0	3	1	0	0
Denmark	7	3	3	0	0	0
Estonia	3	0	1	1	1	0
Finland	8	0	5	3	0	0
Uzbekistan	1	1	0	0	0	0
France	14	0	4	3	1	1
Israel	12	0	8	1	0	1
Georgia	3	0	0	0	0	0
Germany	68	5	35	13	2	1
Kazakhstan	1	0	0	0	0	0
Greece	40	2	17	6	1	0
Hungary	6	1	3	2	0	0
Iceland	1	0	0	0	0	0
Ireland	8	1	5	0	0	0
Italy	30	2	15	3	1	0
Latvia	10	2	6	0	0	0
Lithuania	9	0	5	1	0	0
Luxembourg	1	0	0	0	0	0
Malta	7	1	2	3	0	0
Netherlands	35	16	2	6	1	2
Norway	11	2	3	5	0	1
Poland	15	0	9	0	0	0
Portugal	57	5	23	5	1	0
Republic of Moldova	4	0	2	1	0	0
Romania	25	2	9	5	0	0
Russian Federation	11	0	5	3	0	0
Serbia	31	3	6	5	1	0
Slovakia	3	2	0	0	0	0
Slovenia	7	1	2	1	1	0
Spain	49	2	18	9	1	0
Switzerland	29	2	11	8	1	0
The former Yugoslav Republic of Macedonia	5	0	3	0	1	0
Turkey	58	8	9	21	2	0
Ukraine	7	1	3	1	0	0
United Kingdom of Great Britain and Northern Ireland	32	5	4	12	1	1
Azerbaijan	1	0	0	0	0	0
Sum	710	80	258	138	20	8

More than half of respondents are involved in organizing teaching programs in their institutions, 55.34% (280/506).

### Actual knowledge of competence-based curricula

Regarding the current educational program of the specialty, most respondents claim that there is one curriculum on the national level in their countries (426/546; 78.02%). Smaller number claim there is not (57/546, 10.43%) or they do not know (63/546; 11.54%). In some countries the responses are close to be uniform: in Portugal 38/57 know that there is a national curriculum, while only 2/57 do not know, still there are 17 answers missing. In Netherlands (29/35) and in Israel (11/12) claim that there is a national curriculum and there are no other options chosen. Some countries have very conflicting responses: in Spain, 24/49 respondents declare knowledge that there is, 7/29 that there is not and 6/29 do not know if there is national curriculum. In Germany most (40/68) respond positively to the existence of the national curriculum, still 12/68 and 5/68 respond that there is no, or they do not know if there is one, respectively.

Only around a third (203/547; 37.11%) of respondents confirm that they are well-informed about CBMET, a quarter report some knowledge (134/547; 24.59%) and another quarter, no knowledge or not knowing much (47/547; 8.59% and 80/547; 14.62%). Some respondents are convinced that they have knowledge, but CBMET is not implemented in their countries (83/547; 15.17%).

**Figure 3:**
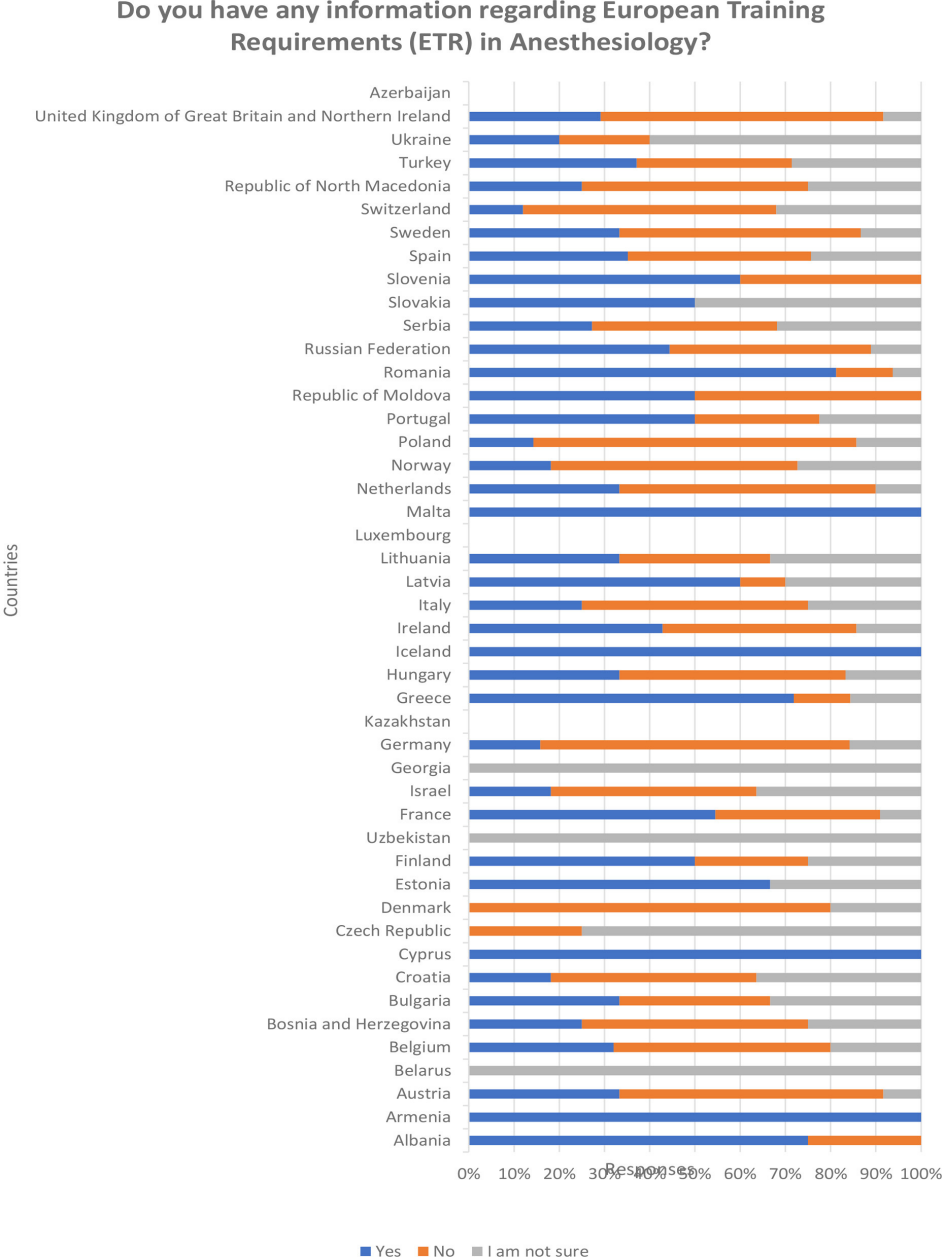
Knowledge of the European Training Requirements (ETR) in Anesthesiology: distribution of answers by the countries.

Most respondents that answer the question regarding having information about ETR in anaesthesiology declared either no knowledge or that they are not sure (232/546; 42.49% and 115/546; 21.06%), while only 36.45% (199/546) claimed that they are informed (Table 5). Mainly the positive answers come from a few countries out of 46: Belgium (8/30), Greece (23/40), Netherlands (10/35), Portugal (20/57), Romania (13/25), Spain (13/49), and Turkey (13/58). However, a disparity between the answers is present regarding ETR knowledge too (Figure 3). Almost the same countries with the highest numbers of respondents declaring knowledge of ETR have high representations of those who have no knowledge at all: Belgium (12/30), Netherlands (17/25), Portugal (11/57), Spain (15/49) and Turkey (12/58) (Table 5).

**Table 5:** Knowledge of the European Training Requirements (ETR) in Anesthesiology: distribution of answers by the countries.

**Do you have any information regarding European Training Requirements (ETR) in Anesthesiology?**	**Number of respondents**	**Yes**	**No**	**I am not sure**
Albania	6	3	1	0
Armenia	1	1	0	0
Austria	15	4	7	1
Belarus	1	0	0	1
Belgium	30	8	12	5
Bosnia and Herzegovina	4	1	2	1
Bulgaria	3	1	1	1
Croatia	15	2	5	4
Cyprus	4	2	0	0
Czech Republic	5	0	1	3
Denmark	7	0	4	1
Estonia	3	2	0	1
Finland	8	4	2	2
Uzbekistan	1	0	0	1
France	14	6	4	1
Israel	12	2	5	4
Georgia	3	0	0	1
Germany	68	9	39	9
Kazakhstan	1	0	0	0
Greece	40	23	4	5
Hungary	6	2	3	1
Iceland	1	1	0	0
Ireland	8	3	3	1
Italy	30	5	10	5
Latvia	10	6	1	3
Lithuania	9	3	3	3
Luxembourg	1	0	0	0
Malta	7	6	0	0
Netherlands	35	10	17	3
Norway	11	2	6	3
Poland	15	1	5	1
Portugal	57	20	11	9
Republic of Moldova	4	2	2	0
Romania	25	13	2	1
Russian Federation	11	4	4	1
Serbia	31	6	9	7
Slovakia	3	1	0	1
Slovenia	7	3	2	0
Spain	49	13	15	9
Sweden	17	5	8	2
Switzerland	29	3	14	8
The former Yugoslav Republic of Macedonia	5	1	2	1
Turkey	58	13	12	10
Ukraine	7	1	1	3
United Kingdom of Great Britain and Northern Ireland	32	7	15	2
Azerbaijan	1	0	0	0
Sum	710	199	232	115

Only 138/428 (33.24%) of respondents reply that an outcome-based curriculum is implemented in their hospital. The highest numbers came from the Netherlands (20/35) and the UK (17/32). Around a third of respondents claim that the curriculum is still content based (case based) and 20 % of them do not even have knowledge of the structure of the educational program in their hospitals.

Mostly, national bodies responsible for education or universities are responsible for curriculum development (229/541 and 72/541; 42.33% and 13.3%, respectively). Professional (anaesthesiology) societies are less often responsible (98/541 or 18.11% of responses). Still, there is no consensus among respondents within the countries regarding this question too (for example Germany: 17/68 national body, 24/68 departments of anaesthesiology, 6/68 society, and 7/68 do not know).

It is clear from responses that most residents must pass the national exam (282/542 or 52.03%) or the university exam (92/542 or 17.01%) to become specialists. Some respondents say that just finishing the residency program qualifies residents for specialty (81/542, answers from France, Italy, Norway, Spain, Sweden, and Turkey). EDAIC part I (European Diploma of Anaesthesiology and Intensive Care) is present in some countries as a part of a specialty exam (Austria, Belgium, Hungary, Netherlands, Romania, Sweden, and Switzerland).

Structured exam (oral exam or VIVA, written exam, and OSCE) is present only in few countries (49/497 and 16/497; 9.86% and 3.21%): Belgium, Israel, Ireland, Netherlands, Ukraine, and UK. Many residents must pass either an oral exam (143/497; 28.77%) or an oral and written exam (145/497; 28.18%) to qualify as a specialist.

## Discussion

The results of this survey have shown that among experienced specialists involved in training and teaching anesthesia only one-third had knowledge of CBMET and are informed about the ETR document and its content. Formal training in teaching has only one-fifth of the respondents and a few countries have obligatory courses for teaching their educators at universities (Germany, Netherlands, Turkey, and UK). More than half of the respondents claim that their work in teaching is not valued and is considered a part of everyday work. Lack of time and overload with work are reported as the most important obstacles in performing teaching to residents.

The value of the ETR document is the guidance for implementing competence-based curricula in different countries (2). It is a document based on the changed paradigm of medical education and achieving those objectives could be the common ground for standardization of training in various countries.

Our survey recovered that even experienced specialists involved in delivering training and mentoring residents lack knowledge of the theory behind the new concepts and skills necessary to deliver it. Lack of knowledge is present even for the structure of the formal documents and recommendations in implementation of the CBMET in local, state, or European level. This may be the reason for the slow change of the training programs of anesthesia residency programs in Europe. Recent data show significant variation in the structure of anesthesia residency training programs across Europe, particularly regarding the implementation of CBMET (13). Survey responses collected from the European trainees indicate that United Kingdom and Austria are the only countries where CBMET is fully implemented. In Germany, the Netherlands, Norway, and Sweden, while training is largely competency-based, it still includes rotations of specific duration and a minimum number of cases. In Belgium, the Czech Republic, and Poland, the training is primarily case-based, though some CBMET-based rotations are included. In the remaining countries, training continues to be predominantly case-based. This newer data strongly aligns with findings from our survey of the trainers, reinforcing the consistency of perspectives between these two groups. Respondents in our survey who reported the highest levels of familiarity with CBMET and ETR are from countries that, according to trainees, are also experiencing a positive transition to CBMET. It appears that sets of responses, both from trainees and trainers, indicate that the transition to CBMET is ongoing but uneven across Europe, with certain countries integrating CBMET elements more fully than others.

Respondents in our survey claim that in most countries national bodies and professional societies are responsible for the curriculum development. It would be informative to know how much knowledge on ETR and standards of education and patient care is spread within those institutions and policy makers.

The driving force in implementing change in medical education, anesthesia and intensive care included, is the faculty (14). Many barriers in faculty development have already been recognized. Increased costs, lack of understanding and poor engagement are just some of them (7). It has been noted before that developing teaching skills should go beyond volunteerism and must be recognized as the place for general improvement and investment (4, 15, 16). Less than 20% of our respondents confirmed that the elements of developing teaching skills (how to teach others) are included in their residency program. However, of greater concern is that most members of the faculty still consider as the main part of their formal education in teaching attendance the ALS courses. A minority of the universities have developed obligatory training in medical education. It may seem that specialists that are actively involved in training residents are mainly left on their own, their internal motivation and self-directed learning to navigate to the contemporary expectations in medical education. Yet, lessons learned from centers that have already reformed training are that significant financial and human resources are needed. It is argued that support and recognition of the teachers' effort in teaching, observing, and giving feedback should be as equal to doing research (4). Faculty development is a long and slow process at both system and individual front-line teachers' levels (14). A consistent, systematic approach toward faculty development is necessary, but it seems that it is still absent in many European countries and institutions.

The lack of time and overload with work seem to be the most rated obstacles to involvement in teaching in this survey. It seems that teaching is just an add on to the everyday work, and that additional time for teaching and preparing for teaching must be over and above working hours. The question of motivation to become more educated for or to deliver high quality teaching is not easy to answer. Conflicting answers regarding the value of teaching and different level of formal training in teaching that come within the countries can be explained with different practices in different hospitals and regions, as well as different formal teaching positions. However, it still reflects diversity in the organization of teaching not only between countries, but within countries themselves.

A recently published short scientific report pointed out the need for standardization of training in Europe (17). The focus was placed on existing differences in duration of the training (recommended minimum of 5 years) and the lack of standardized final exam, mainly a few countries adopting EDAIC exam as the structured assessment tool. Although this plea for standardization of the training is in line with the ETR document, it is still distinguishing time-based education and summative assessment tools. Although it may be assumed that these elements of education would be the easiest to harmonize, it still does not guarantee comparable levels of training between residents and young specialists. That said, it is important to note that the duration of anesthesia residency training currently varies significantly across countries, ranging from 24 to 84 months. Over 40% of European countries still do not meet the training duration of 60 months (13). A significant difference in the structure of training persists, particularly regarding intensive care training.

In this survey, we did not explore the implementation of specific competencies as defined by the ETR or other professional societies, such as the European Society of Intensive Care Medicine (ESICM). Instead, we focused on whether specialists who are trainers are aware of the existence of such documents or are knowledgeable of the information they convey. A comparison of the current training structures with data on the implementation of CBMET from our survey suggests that the paradigm shift is slow (13). This delay may be attributed to the lack of a systemic approach to faculty development.

Additionally, quite recently an electronic survey among the National Anesthesia Societies Committee members (NASC) was conducted. NASC is the part of the ESAIC and is consistent of representatives of each national society of the 41 European country members (18). A questionnaire was designed to investigate the organization of training in anaesthesiology and intensive care medicine within the countries according to the knowledge of the representatives. It included 10 questions, 7 focused on the main changes introduced in the 2022 ETR in anaesthesiology. It was reported that EPAs have been introduced in 34% (13/41) of the countries, 41% have national standardized electronic portfolio offered to the trainees, and almost half of the countries (46%) have not organized any initiative regarding the implementation of the ETR. The authors conclude that further efforts and potentially increased investments are warranted to make this transformative change. We believe that while the respondents in the published survey included national representatives, our results emphasize that there is a significant diversity in the organization of teaching not only between countries, but within countries themselves. Looking at individual responses from the anaesthesiologists we may assume that within the country, even if CBMET present, incoherent practice may be expected at different hospitals or universities. Still, it seems that the similar results are replicated, coming from different angles.

Although most countries require an exam at the end of training (50% national and 17% university board), only around 30% have either structured (oral exam-Viva, written and objective structured clinical examination–OSCE) or some kind of practical assessment. Strikingly, there are still countries where finishing the residency program without examination is qualifying for the specialist. It is therefore not possible to compare or objectively determine what level of competences, what skills and knowledge have been achieved at the end of the training.

In the survey published in 2017 among UEMS EBA country representatives, information about the assessment and certification process within the European countries was explored (19). A great diversity in assessment and certification processes in European anesthesia training has been shown, with very few countries adopting CBMET certification process at the time. It was difficult to strictly categorize countries and majority of them were still in the apprentice model of training. At the time 6 countries included EDAIC exam in the assessment and certification process. Our results have shown that only one additional country adopted EDAIC exam in the meantime (difference of 6 years in collecting results). Other results regarding assessment do not witness any substantial change. Implementation of CBMET in training reflects in the methods of assessment and explicitly defined competences as well as introducing EPAs (19). If they are not existing in the assessment and certification, we may assume that little change toward CBMET took place. Again, our survey demonstrates a lack of improvement of the assessment methods and change toward the new concept within recent years. We believe that this is a result of the fact that trainers and teachers are not trained for the new methods of assessment and are not systematically involved in CBMET implementation.

Most respondents in this survey value and have an interest in teaching. But even they are with the lack of knowledge and information about recommended requirements for training in anesthesia and intensive care in Europe. If training is happening every day in operating theaters and intensive care units, while working with more senior doctors as a part of their everyday job, we may question the structure of that training if the faculty itself is so diverse. We can see from different perspectives that standardization through implementation of the ETR in anaesthesiology is slow and challenging process (13, 18). That seems very convincing that change cannot be achieved without the trainers that are trained in what they should perform. Standardization of training comes from the outcomes of education that should be structured and delivered according to the recommended requirements. Substantial improvements and change of practice in education will not happen without investing in training the faculty and recognizing teaching as one of the competencies of the highest priority in our profession.

### Limitations

The main limitations of this survey are the availability of the questionnaire in English only and the low response rate. Language barriers may have posed challenges for the respondents from non-native English-speaking countries, potentially affecting accuracy of the responses. Regarding the response rate, however, we did receive responses from anesthetists from almost all European countries. Although, it may still not reflect the real cross-section of the current teaching practices and knowledge related to medical education, we believe that the results of this study highlight the differences within and between European countries regarding the perception about CBMET. Recent published data related to the similar objective (13, 18) appear to be in line with our results, indicating that responses collected in this survey reflect the current status of the European anesthesia faculty development.

## Conclusion

If the accepted training requirements in anesthesia and intensive care and future harmonization of the acquired competencies of young specialists in Europe are going to be implemented, faculty development represents one of the initial key steps. The results of the presented survey reveal that even among experienced anesthesia professionals dedicated to medical education there is still a lack of knowledge on CBMET as well as systemic support for faculty development in European countries. The enthusiasm of individuals cannot be the only driving force in changing the paradigm of education. Clear objectives in implementing the CBMET should be established, plans executed, and teaching must become valued to improve anesthesia training and ultimately standards of care among European countries.

## Data Availability

The original contributions presented in the study are included in the article/Supplementary material, further inquiries can be directed to the corresponding author.

## References

[1] Van Gessel E Mellin-Olsen J Østergaard HT Niemi-Murola L. Education and training standing committee, European board of anaesthesiology, reanimation and intensive care. Postgraduate training in anaesthesiology, pain and intensive care: the new European competence-based guidelines. Eur J Anaesthesiol. (2012) 29:165–8. 10.1097/EJA.0b013e32834da759 22418836

[2] EBA ETR. (2022). UEMS. Training Requirements for the Specialty of Anaesthesia, Pain and Intensive Care Medicine. Available at: https://drive.google.com/file/d/1r2dlzJiPvM0SVM2hBrK6m4ZeNV1i68ue/view/ (accessed January 12, 2025).

[3] Cumming A Ross M. The Tuning Project for Medicine–learning outcomes for undergraduate medical education in Europe. Med Teach. (2007) 29:636–41. 10.1080/01421590701721721 18236249

[4] Nousiainen MT Caverzagie KJ Ferguson PC Frank JR ICBME Collaborators. Implementing competency-based medical education: what changes in curricular structure and processes are needed? Med Teach. (2017) 39:594–8. 10.1080/0142159X.2017.1315077 28598748

[5] Wang A. Review article: teaching, learning, and the pursuit of excellence in anesthesia education. Can J Anaesth. (2012) 59:171–81. 10.1007/s12630-011-9636-x 22135210

[6] Harden RM CJ. AMEE Guide No 20: the good teacher is more than a lecturer - the twelve roles of the teacher. Med Teach. (2000) 22: 334–34. 10.1080/014215900409429

[7] Fraser AB Stodel EJ Jee R Dubois DA Chaput AJ. Preparing anesthesiology faculty for competency-based medical education. Introduction de la formation médicale fondée sur les compétences en anesthésiologie: comment préparer le corps professoral? Can J Anaesth. (2016) 63:1364–73. 10.1007/s12630-016-0739-2 27646528

[8] Ebert TJ. Competency-based education in anesthesiology: history and challenges. Anesthesiology. (2014) 120:24–31. 10.1097/ALN.0000000000000039 24158052

[9] Harris DL Krause KC Parish DC Smith MU. Academic competencies for medical faculty. Fam Med. (2007) 39:343–50. 17476608

[10] Sterkenburg A Barach P Kalkman C Gielen M ten Cate O. When do supervising physicians decide to entrust residents with unsupervised tasks? Acad Med. (2010) 85:1408–17. 10.1097/ACM.0b013e3181eab0ec 20736669

[11] Kogan JR Conforti LN Bernabeo EC Durning SJ Hauer KE Holmboe ES. Faculty staff perceptions of feedback to residents after direct observation of clinical skills. Med Educ. (2012) 46:201–15. 10.1111/j.1365-2923.2011.04137.x 22239334

[12] Weller JM Sullivan M Boland J. Does variable training lead to variable care? Br J Anaesth. (2017) 119:866–9. 10.1093/bja/aex265 29028913

[13] Abramovich I Crisan I Sobreira Fernandes D De Hert S Lukic A Norte G. Anaesthesia training designs across Europe: a survey-based study from the trainees committee of the European Society of Anaesthesiology and Intensive Care. Rev Esp Anestesiol Reanim (Engl Ed). (2024) 71:427–37. 10.1016/j.redare.2024.04.006 38636795

[14] Dath D. The importance of faculty development in the transition to competency-based medical education. Med Teach. (2010) 32:683–6. 10.3109/0142159X.2010.500710 20662581

[15] Steinert Y Mann K Centeno A Dolmans D Spencer J Gelula M. A systematic review of faculty development initiatives designed to improve teaching effectiveness in medical education: BEME Guide No. 8. Med Teach. (2006) 28:497–526. 10.1080/01421590600902976 17074699

[16] Hawkins RE Welcher CM Holmboe ES Kirk LM Norcini JJ Simons KB. Implementation of competency-based medical education: are we addressing the concerns and challenges? Med Educ. (2015) 49:1086–102. 10.1111/medu.12831 26494062

[17] Scudellari A Bubenek S Goldik Z Bilotta F. A plea for standardisation in the duration of training in anaesthesiology and intensive care medicine across Europe: a survey of representatives of the European National Anaesthesia Societies Committee. Eur J Anaesthesiol. (2022) 40:138–40. 10.1097/EJA.0000000000001789 36514804

[18] Scudellari A Bilotta F. Standardisation of training in anaesthesiology in Europe: a survey on the impact of the 2022 European Training Requirements in Anaesthesiology. Br J Anaesth. (2024) 133:1104–7. 10.1016/j.bja.2024.07.029 39327152

[19] Jonker G Manders LA Marty AP Kalkman CJ Ten Cate TJ van Gessel EF. Variations in assessment and certification in postgraduate anaesthesia training: a European survey. Br J Anaesth. (2017) 119:1009–14. 10.1093/bja/aex196 28981584

